# Editorial: Case reports in veterinary neurology and neurosurgery, volume II

**DOI:** 10.3389/fvets.2026.1964788

**Published:** 2026-09-18

**Authors:** Koen M. Santifort, Joao Miguel De Frias, Simone Spinillo, Bruno A. Lopes, Susana Monforte Monteiro

**Affiliations:** 1 IVC Evidensia Small Animal Referral Hospital Arnhem, Neurology, Arnhem, Netherlands; 2 IVC Evidensia Small Animal Referral Hospital Hart van Brabant, Neurology, Waalwijk, Netherlands; 3 Hospital for Small Animals, Royal (Dick) School of Veterinary Studies, The University of Edinburgh, Edinburgh, United Kingdom; 4 ChesterGates Veterinary Specialists, Chester, United Kingdom; 5 Southfields Veterinary Specialists, Part of Linnaeus Veterinary Limited, Basildon, United Kingdom; 6 Department of Veterinary Medicine, University of Cambridge, Cambridge, United Kingdom

**Keywords:** cat, dog, fox, neurology, neurosurgery

In the ongoing pursuit of knowledge, case reports provide clinicians in veterinary medicine with useful and clinically relevant information. In the first edition of this research topic “*Case Reports in Veterinary Neurology and Neurosurgery—Volume I*,” 17 articles were included that covered a wide range of subjects, including clinical phenomena, histopathological diagnoses, genetic disorders, and other.

In this second volume (*Case Reports in Veterinary Neurology and Neurosurgery, Volume II*), no less than 24 articles are included. Of these, nine pertain to cats, one to foxes, and 14 to dogs. The number of case reports pertaining to cats is interesting to note, as much of what is known in veterinary neurology about cats comes from either studies with a relatively low number of patients or case series and reports.

## Cats

In their publication titled “*Case Report: Feline spinous process giant-cell osteosarcoma*,” Vercoutere et al. reported two cases of vertebral giant cell osteosarcoma in cats with progressive myelopathy with clinical findings, diagnostic imaging, surgery, follow-up, and histopathology. This differential diagnosis is important to consider when cats present with similar clinical and diagnostic imaging findings and can help guide prognostication and treatment options. Interestingly, the cat was suspected to have a C1-5 myelopathy based on clinical findings of upper motor neuron hemiparesis, but a dorsally located mass was found at the level of the C6 vertebra. Authors speculate that the dorsal localization may have resulted in a lack of lower motor neuron signs of the thoracic limbs.

Reports on intervertebral disc disease in cats are much less common than in dogs where the pathology, clinical signs, treatment options, and outcomes are well-described. In their report titled “*Case Report: Two adjacent ventral slots for treatment of multiple compressive cervical intervertebral disc protrusions in a British Shorthair cat*,” Castellano and Mól describe the application of a surgical technique previously described in dogs in a cat with cervical intervertebral disc disease.

Muller et al. report the surgical treatment of a lumbar meningomyelocele in a 2.5 year-old cat in their publication titled “*Case Report: Surgical management of a meningomyelocele in a cat*.” As early surgical intervention in indicated in human medicine for neural tube defects, and with the history of chronic paraparesis, incontinence, and spinal hyperesthesia, this surgical option was opted for in this case, though authors describe a mixed outcome in this single case: spinal hyperesthesia was no longer present, although the cat remained non-ambulatory paraparetic with intact deep pain perception and urinary incontinence.

Another surgery-focused article by Hall et al. titled “*Successful surgical management of a cerebellar subdural empyema in a domestic cat*” details the clinical and diagnostic imaging findings, and surgical management of a cat with an intracranial empyema. The authors highlight the importance of considering otitis media as a potential source of intracranial infections in feline patients.

After the advances in the treatment of feline infectious peritonitis (FIP) in the recent years, veterinary neurologists are increasingly presented with FIP-survivors that still have or develop neurological signs. In the report titled “*Post feline infectious peritonitis progressive hydrocephalus: a case series*,” Clouse et al. highlight potential long-term complications of neurological FIP and their management in a small number of cases. Future reports may provide more detail on these phenomena but the report of these cases may provide internal medicine specialists, general practitioners, and neurologists with important information to consider in their FIP patients.

With their publication titled “*Case Report: Presumptive spinal embryonal tumor in a cat*” van den Heuvel et al. extensively characterized a rare spinal neoplasia in a cat including clinical characteristics and diagnostic imaging, surgical, and histopathological findings. Just recently before this publication, another case of a feline spinal embryonal tumor was reported (1); these two reports both contribute important information for these tumor types to be considered as a differential diagnosis based on diagnostic imaging, surgical, and histological findings including extensive immunohistochemistry tests.

In “*Case Report: Generalized motor tonic seizures characterized by paroxysmal fast activity on EEG in a Tonkinese cat*” Brewinska et al. document tonic (dystonic) motor seizures with a generalized onset confirmed by electroencephalography in a cat. Treatment with phenytoin was reported to improve quality of life and seizure control in this single case.

With extensive characterization of diagnostic imaging, surgical, histological and post-mortem findings, Vezza et al. in their report titled “*Case Report: Primary intracranial lymphoma and meningioma manifesting as a composite tumor in a cat*” describe a clinically relevant possibility of a composite tumor (two types of neoplasia colliding in one patient) in small animal neurology and its potential implications for case management and outcome.

Infectious causes for myelopathies in small animals remain an important differential diagnosis even though autoimmune diseases are prevalent. In “*Case Report: Mycobacterial epidural pyogranulomatous steatitis in a cat*,” Decrop et al. detail the possibly difficult and misleading diagnostic path to obtaining a confirmed diagnosis of mycobacterial infections and emphasize that such infections should be considered a differential diagnosis in cases of pyogranulomatous epidural pathology, even in the absence of systemic dissemination.

## Dogs

In the publication titled “*Case Report: Surgical resection of high-grade extradural thoracic vertebral chondrosarcoma in a dog*,” Ho et al. describe the clinical presentation, diagnosis, treatment, and outcome of a dog with a thoracic vertebral chondrosarcoma. Interestingly, this dog presented with an acute onset of clinical signs consisting mostly of hyperesthesia or “pain.”

Kamp et al. extensively describe their diagnostic imaging and histopathological findings in their report titled “*Case Report: Segmental spinal cord hypoplasia in a Labrador Retriever puppy*.” The conspicuous MRI finding of an enlarged subarachnoid cavity, especially dorsally to the spinal cord, with a spinal cord: dorsal subarachnoid space ratio of about 50% raised the suspicion of hypoplasia. The detailed report of the clinical findings, diagnostic imaging, and post-mortem observations can help to identify similarly affected cases in the future.

The diagnosis of hemangiosarcoma in dogs carries poor prognoses in general, especially when this type of neoplasia affects the brain. However, obtaining an *in vivo* confirmation of the diagnosis is, when the brain is affected, troublesome. In their case report titled “*Surgical treatment and outcome of intracranial hemangiosarcoma in two dogs: case series,”* Biundo et al. detail their findings and approach to two canine cases with intracranial hemangiosarcoma, including surgical treatment and adjunctive treatment, and follow-up. The patient treated with adjunctive therapies had a longer post-operative survival time than the patient treated with surgery alone (87 vs. 314 days).

Treatment options for malignant nerve sheath tumors in dogs are increasingly reported, though still often limited to surgery including amputation and/or radiation therapy. In their report titled “*Case Report: Partial neurectomy and limb-sparing treatment for SOX-10 expressing epithelioid malignant nerve sheath tumour of the tibial nerve*,” Spencer-Taylor et al. report their approach to a distal tibial nerve sheath tumor in a dog with lameness. A limb-sparing surgery (neurectomy) resulted in good long-term outcome.

Meij et al. in their publication titled “*Case Report: Treatment of hypersomatotropism in a diabetic dog with transsphenoidal hypophysectomy*” add information to the literature regarding the diagnosis, treatment, and outcome of a dog with hypersomatotropism and diabetes mellitus associated with a pituitary somatotroph adenoma, including endocrinological characteristics. As this is only the second report that describe the treatment of such a tumor and its endocrinological consequences, this report adds significant detail and information of clinical relevance for future patients suspectedly diagnosed with such tumors based on laboratory and diagnostic imaging tests.

Positioning head tilt as a clinical sign has been reported in various neurological disorders in dogs thus far. In the article titled “*Case Report: Positioning head tilt observed in six dogs with meningoencephalitis of unknown origin*,” Tamura et al. describe the observation of this clinical sign in dogs with meningoencephalitis of unknown origin. Although in all of these cases the midbrain was involved, the occurrence of multifocal lesions prohibits firm conclusions as to which lesion of which neuroanatomical structure(s) was or were the cause of this clinical sign. Nevertheless, the authors stress that the observation of this clinical sign has not been reported to be caused by potentially acute, progressive and lethal disorders such as meningoencephalitis before and that this disorder should be considered as a differential diagnosis in pertinent cases.

On a similar note, Mendo Reyes et al. documented a conspicuous clinical sign of a ‘dropped head' in two dogs in their publication titled “*Case Report: Dropped head syndrome as a main clinical sign of suspected myasthenia gravis in two dogs*.” Although definitive confirmation of the diagnosis was lacking, the clinical characteristics and outcomes following treatment suggest that this differential diagnosis is relevant to consider in similarly presenting cases in the future.

Babenko et al. describe the use of an individualized 3D printed mold for a polymethylmethacrylate plate cranioplasty in a dog in their report titled “*Novel approach to a severe craniomaxillofacial trauma in a military working dog using a customized 3D-printed mold for printed polymethylmethacrylate (PMMA) plate–a case report*.” For complex reconstructive surgery, this report adds clinical considerations and experience to take into account for the management of future cases similar to this.

In the report titled “*Case Report: 3D-CISS and PC-MRI in the diagnosis and surgical planning of hydrocephalus secondary to presumptive lateral aperture obstruction in a dog*” Tauro et al. emphasized the importance of the application of specific MRI sequences for considering optimal treatment of canine hydrocephalus and syringomyelia.

While intervertebral disc disease is so well-reported in canine veterinary neurology literature, this multifaceted disorder still does not cease to provide clinicians with diagnostic challenges. In the publication titled “*Case Report: Dural infiltration of intervertebral disc material mimicking spinal cord meningioma in a dog*” a new meaning is added to the term “intradural” as authors describe their findings and treatment for a dog with a not previously reported presentation of an intervertebral disc herniation (Sakamoto et al.).

Bellomo et al. published their report titled “*Case Report: Clinical and MRI features of hemorrhagic transformation of an ischemic cerebrovascular accident in a dog*” that details the development and imaging findings of an ischemic cerebrovascular that transformed to become hemorrhagic; a severe and potentially lethal complication. The dog in this report was euthanized due to this development.

In the report titled “*Case Report: Suspected “stiff dog syndrome” in a Maltese dog*” Buffagni et al. describe a rare clinical case exhibiting features comparable to those of stiff person syndrome in humans and stiff horse syndrome in horses—similarly rare neurological disorders affecting these other species. This syndrome was characterized by generalized hypertonicity of the axial and proximal appendicular muscles with superimposed episodic muscle spasms. The diagnosis was based on electromyographic studies and the presence of glutamic acid decarboxylase antibodies in serum of the patient.

Liatis et al. published the report titled “*Case Report: Phenotypic and genetic characterization of a presumptive sporadic hypothalamic hamartoma in a standard Schnauzer dog*” with clinical and diagnostic imaging characterizations in addition to genetic testing. While the authors acknowledged limitations to the diagnosis in this case, the occurrence of continuous repetitive involuntary movement of the head and neck in this dog with a lesion predominantly affecting the hypothalamus and adjacent structures is noteworthy to consider for future cases of involuntary movements in dogs.

Antes et al., in their publication titled “*Case Report: Hypodipsic hypernatremia secondary to hydrocephalus in a dog*” detail dysregulation of sodium homeostasis in a dog with severe hydrocephalus. The authors hypothesized that “*hypodipsic hypernatremia in this case*—was secondary to—*pressure necrosis of the hypothalamus secondary to worsening of congenital hydrocephalus, leading to dysfunction of the lamina terminalis and its control over the generation of thirst sensation*.”

## Other

With meningoencephalitis of unknown origin documented so well in veterinary neurology literature for dogs and less so in cats, reports in yet other species can significantly contribute to the ability to diagnose and potentially treat this disorder in future patients other than cats and dogs. In their report titled “*Case Report: Meningoencephalomyelitis of unknown origin in foxes (Vulpes vulpes and Vulpes lagopus): insights into the diagnostic challenges in carnivore neurology*,” authors did exactly that, characterizing their attempts to identify pathogens and failing to do so (Steiner et al.). Perhaps, the disorder encountered in these two foxes is indeed similar to what has been reported in various dog breeds and a number of cats thus far.

Each case report included in this second volume contributes to our understanding or hypothesis-generation of numerous pathological phenomena. The next volume https://www.frontiersin.org/research-topics/77904/case-reports-in-veterinary-neurology-and-neurosurgery-volume-iii is open for submissions and the editorial teams invites contributions.
